# Impact of Maternal Ultra-Processed Food Consumption and Preterm Birth on the Development of Metabolic Disorders in Offspring

**DOI:** 10.26502/jppch.74050214

**Published:** 2025-04-28

**Authors:** Gauri Gurumurthy, Devendra K. Agrawal

**Affiliations:** Department of Translational Research, College of Osteopathic Medicine of the Pacific, Western University of Health Sciences, Pomona, California, 91766 USA

**Keywords:** Fetal development, Fetal programming, Maternal nutrition, Metabolic disorders, Preterm birth, Ultra-processed foods

## Abstract

This review examines the growing concern regarding the relationship between maternal ultra-processed food (UPF) consumption, preterm birth, and the subsequent development of metabolic disorders in offspring. Ultra-processed foods have become increasingly prevalent in global diets, coinciding with rising rates of metabolic diseases. Concurrently, preterm birth remains a significant public health concern affecting 5–18% of births worldwide. Here, we critically reviewed the current evidence regarding how maternal UPF consumption affects fetal development and how preterm birth disrupts metabolic programming. Furthermore, the information is presented on the potential synergistic effects when both factors are present. The mechanisms underlying these associations, including fetal malnutrition, inflammation, and hormonal dysregulation, are analyzed. Research suggests that maternal UPF consumption and preterm birth independently contribute to altered metabolic health in offspring, with potential compounding effects when both factors are present. This review highlights the public health implications of these findings and identifies areas requiring further research to better understand the complex interplay between maternal diet, preterm birth, and long-term metabolic health outcomes in offspring.

## Introduction

### Ultra-Processed Foods (UPFs)

Ultra-processed foods (UPFs) have become increasingly prevalent in modern diets globally, leading to growing concerns about their impact on public health. According to the NOVA food classification system, UPFs are defined as industrially manufactured foods made predominantly from substances extracted from foods and chemically modified, often with the addition of various additives [1]. These typically include sugary snacks, packaged meals, refined grains, and other convenience foods characterized by high energy density and low nutritional value.

The global consumption of UPFs has risen dramatically in recent decades, particularly in Western countries. In the United States, total UPF intake is substantially higher compared to Europe, with specific subgroups such as ultra-processed breads, cereals, and artificially or sugar-sweetened beverages making up a larger portion of the diet [2]. This dietary shift has coincided with increasing rates of obesity, diabetes, and other metabolic diseases worldwide.

Despite the known association between UPFs and adverse health outcomes, definitive evidence directly linking their consumption to obesity and other diseases has been evolving. Recent controlled studies, including those from the National Institutes of Health (NIH), provide compelling evidence that diets composed primarily of UPFs lead to higher calorie consumption and weight gain, even when sugar, fat, fiber, and macronutrient composition are matched with unprocessed diets [3,4]. These findings suggest that there may be intrinsic properties of UPFs beyond their nutritional composition that contribute to adverse health outcomes.

### Overview of Preterm Birth

Preterm birth, defined as birth before 37 weeks of gestation, remains a significant global health concern. The prevalence of preterm births ranges from 5% to 18% across different countries, with higher rates typically observed in developing nations [5]. A study found that the leading cause of mortality in children under five is pre-term birth complications making up 16.6% of all neonatal deaths worldwide [6].

According to NIH report in 2023, preterm births remained relatively stable in the US with a marginal increase from 10.38% in 2022 to 10.41% in 2023, though showing a slight 1% decrease from 2021 levels (10.49%). Early term births demonstrated a notable upward trend, increasing by 2% from 29.31% in 2022 to 29.84% in 2023, and reflecting a more substantial 4% rise since 2021 (28.76%) [7]. This shows that although USA is a developed nation, the number of preterm and early term births are still significant, highlighting the importance of understanding the effects on infants born preterm.

The etiology of preterm labor is complex and multifactorial. Current understanding suggests that preterm birth results from a combination of factors including infection, cervical pathology, uterine overdistension, progesterone deficiency, maternal and fetal stress, allograft reactions, and allergic phenomena. Preterm premature rupture of membranes (PPROM) is a significant cause, often preceded by asymptomatic intrauterine infections. The exact causes of preterm labor are unclear but may involve a combination of factors like fetal cortisol production, progesterone withdrawal, and uterine contractions triggered by oxytocin. The fetal-adrenal axis and the imbalance between estrogen and progesterone also play crucial roles in initiating labor. Additionally, there may be unknown factors contributing to the onset of preterm labor [5].

While advances in neonatal care have significantly improved survival rates for preterm infants, these children face higher risks of developing various long-term health complications. These include respiratory conditions such as bronchopulmonary dysplasia, gastrointestinal issues like necrotizing enterocolitis, and cognitive and developmental delays. Moreover, preterm birth has been associated with metabolic health issues in later life, including increased risks of obesity, diabetes, and cardiovascular disease, as early birth disrupts normal development and the establishment of metabolic systems [8].

### The Importance of Maternal Diet in Fetal Development

The maternal diet during pregnancy plays a critical role in shaping fetal development and long-term health outcomes in children. Nutritional factors, including macronutrient balance, micronutrient sufficiency, and overall diet quality, influence placental function, fetal growth, and metabolic programming [2,4]. Inadequate maternal nutrition, whether due to undernutrition or consumption of nutrient-poor, energy-dense foods such as UPFs, can have profound effects on fetal development and subsequently increase the risk of various health issues in offspring, including metabolic disorders.

A study examined the associations between maternal dietary patterns during the second and third trimesters of pregnancy and fetal growth, specifically focusing on head circumference. The results indicated that diets rich in animal products were positively associated with fetal head circumference. In contrast, vegetable-heavy dietary patterns were linked to a reduction in fetal head circumference, with the latter also increasing the risk of small head circumference for gestational age. Notably, adhering to the same dietary pattern across both trimesters showed stronger effects on fetal growth. The study highlighted that maternal diet influenced head growth more significantly than other growth indicators such as abdominal circumference (AC), femur length (FL), or birth weight [9].

The relationship between maternal diet, preterm birth, and offspring metabolic health represents a complex interplay of factors that have significant public health implications. This review aims to explore the current evidence regarding how maternal UPF consumption affects fetal development, how preterm birth disrupts metabolic programming, and the potential synergistic effects when both factors are present.

## Impact of Maternal Ultra-Processed Food Consumption on Offspring Metabolic Health

### Influence of Maternal UPF Consumption on Fetal Development

Maternal consumption of nutrient-poor, energy-dense foods, such as those typical ultra-processed foods (UPFs), can significantly impact fetal development through various mechanisms. The concept of fetal programming suggests that maternal nutritional imbalances during pregnancy can influence the metabolic development of the child, potentially predisposing them to metabolic disorders later in life [10]. UPFs, which are often high in added sugars, trans fats, and low in essential micronutrients, can disrupt the optimal nutritional environment required for proper fetal growth and development.

A study investigated the impact of maternal dietary patterns, particularly a mixed, high-sugar diet, on fetal growth, finding that adherence to this diet was linked to increased fetal measurements, including biparietal diameter, head circumference, abdominal circumference, and femur length. Notably, these effects were specific to male fetuses, highlighting a potential sexual dimorphism in the response to maternal diet. The study also underscores the role of maternal diet in the growing concerns of fetal overgrowth and its long-term implications, particularly in populations at risk of gestational diabetes and obesity. While maternal body mass index (BMI) and weight gain were not shown to alter these associations, the study advocates for improved preconception and prenatal care, focusing on healthier dietary habits to optimize both maternal and fetal health [11].

Similarly, a large cohort study conducted in Europe found that a lower quality, more pro-inflammatory maternal diet was linked to smaller birth sizes, including lower birth weight and shorter birth length, and an increased risk of delivering small-for-gestational-age (SGA) infants, independent of pre-pregnancy BMI and socioeconomic factors. Notably, the study observed sex differences in how maternal dietary inflammatory potential affected birth outcomes. The results were consistent across various sensitivity analyses, including those focusing on healthier mothers without pregnancy complications and full-term infants. Adjusting for gestational age had little impact on the findings, indicating that the relationship between maternal diet and birth size was not mediated by gestational duration. While mutual adjustment for dietary patterns and inflammation reduced the associations, it did not eliminate them entirely, suggesting that other mechanisms, such as epigenetic programming, might also play a role [12].

Both studies independently found that maternal pre-pregnancy BMI and weight gain had a relatively low impact on gestational birth weight and head circumference, while the quality of the diet consumed during pregnancy had a significant effect on fetal growth, with notable sex differences observed. These findings emphasize the crucial role of maternal nutrition during pregnancy, regardless of socioeconomic status, in influencing fetal health. The results suggest that improving access to nutrition and providing education about healthy eating for new mothers is vital, as dietary quality during pregnancy appears to have a more profound impact on fetal development than maternal weight-related factors.

Maternal dietary patterns during pregnancy can significantly alter placental function, influencing nutrient transport, hormone secretion, and fetal development. High intake of trans fats and sugar has been linked to changes in the fetal epigenome, which can lead to altered metabolism and a heightened risk of obesity in offspring. These early developmental changes are associated with long-term health risks, including metabolic diseases, insulin resistance, and obesity in childhood. Such findings further underscore the importance of optimizing maternal nutrition during pregnancy, as it plays a pivotal role in shaping both immediate and future health outcomes for the child [13].

### Role of Key Nutrients in Fetal Development

Key nutrients play essential roles in fetal development, and their deficiencies or imbalances can have significant consequences for the developing fetus. Long-chain polyunsaturated fatty acids (n-3 and n-6), vitamins, and minerals are crucial for optimal fetal development. Deficiencies in these nutrients can impair fetal neurodevelopment, growth, and metabolic function. For example, maternal docosahexaenoic acid (DHA) deficiency has been shown to affect fetal neurodevelopment and alter placental epigenetics, leading to impaired growth and development [14].

Across three studies in different regions of the world, contradicting effects of a high prudent maternal diet on fetal development were observed. A high prudent diet is characterized by a high intake of vegetables, fruits, poultry, fish, whole grains, and foods low in added sugars, unhealthy fats, and processed foods. For instance, a Norwegian study found that women adhering to a high prudent diet had an increased risk of small for gestational age (SGA) and a decreased risk of large for gestational age (LGA) [15], whereas a Danish cohort study, using a similar ultrasound-based definition of SGA, found that women with a health-conscious diet (similar to the high prudent group) had a reduced risk of SGA compared to those following a Western dietary pattern [16]. Similarly, studies from China and India also observed that diets rich in fruits, vegetables, and other nutrient-dense foods were associated with higher birth weight and a lower prevalence of SGA [17,18]. In contrast, a US study involving 1,151 pregnant women and seven different dietary patterns reported no associations between any of the dietary patterns and SGA or LGA [19]. These discrepancies may arise from cultural, geographic, and dietary differences between countries, such as the higher consumption of seafood and cod liver oil in Norway compared to Denmark [15]. Additionally, factors like genetic differences, maternal age, BMI, and the presence of underlying conditions such as gestational diabetes further complicate these associations. The variation in findings across different populations and study designs underscores the need for further research to better understand how maternal diet interacts with these factors to influence fetal growth and birth weight outcomes.

Micronutrients, including vitamins A, D, E, folate, B12, B6, C, iron, zinc, iodine, copper, and selenium, play vital roles in maternal, placental, and fetal health during pregnancy. These essential nutrients support metabolic functions such as cell signaling, growth, and differentiation [20]. While the primary goal is to obtain the necessary micronutrients from food, a diet with low nutritional value makes this extremely difficult, leading for the need for supplementation. Iron during pregnancy is crucial for fetal brain development, with deficiency leading to immediate alterations in brain structure and long-term neurocognitive impairments. Even non-anemic maternal iron deficiency can compromise fetal brain function, particularly in recognition memory. Additionally, inadequate fetal iron stores increase the risk of postnatal iron deficiency and associated neurodevelopmental issues, including motor and social dysfunction [21].

### Alterations in Metabolic Programming

Maternal diet plays a crucial role in shaping the metabolic health of offspring, with exposure to high-fat diets during pregnancy being a significant contributor to the development of insulin resistance and altered lipid metabolism in children. Studies have shown that maternal high fat diet exposure during pregnancy and lactation leads to long-term metabolic disturbances in offspring, including impaired insulin sensitivity, increased adiposity, and hepatic fat accumulation [22].

In adults, excessive consumption of ultra-processed foods (UPFs) is linked to significant metabolic health issues, including excessive weight gain and insulin resistance. UPFs are often energy-dense and rich in refined carbohydrates, sugars, unhealthy fats, and low in fiber and essential nutrients, which disrupt the metabolic processes of the body. Regular consumption of high-glycemic foods and refined sugars can spike insulin levels, leading to insulin resistance over time. This condition impairs the body’s ability to regulate glucose effectively, increasing the risk of developing insulin resistance, type 2 diabetes, obesity, and other features of metabolic syndrome [23–27]. Inflammasomes and gut dysbiosis play a major role in the underlying pathophysiology of diabetes mellitus (28). Indeed, the UPFs may also accelerate the pathogenesis of coeliac disease and decrease the quality of life [29,30].

A randomized controlled trial examined the effects of diets rich in ultra-processed foods (UPFs) on weight gain and insulin resistance. The study found that participants consuming UPFs had significantly higher energy intake and experienced greater weight gain compared to those on unprocessed diets, despite both groups having similar calorie intake. This suggests that the nutritional composition and food quality of UPFs, rather than calorie content alone, contribute to weight gain. Additionally, the increased energy intake from UPFs likely promotes insulin resistance, highlighting the negative metabolic impact of such diets [31].

Similarly, a Brazilian cohort study explored the association between ultra-processed food (UPF) consumption and the development of metabolic syndrome (MetS) over approximately 8 years. The study found that greater UPF consumption (above 552 g/day) was independently associated with a 19% increased risk of MetS, with UPF consumption contributing to 6.6% of new MetS cases. The study also highlights that, even with lower average UPF consumption compared to other populations, increased consumption was linked to a higher risk of MetS, independent of BMI, energy intake, and other dietary factors [32].

Maternal obesity and excessive weight gain during pregnancy are strongly associated with an increased risk of childhood obesity. Studies show that children born to obese women have a 2.5-fold higher risk of obesity at 2–4 years of age compared to those born to non-obese mothers. Additionally, offspring of obese women are more likely to develop obesity and metabolic syndrome by age 11, with a two-fold increased risk. A follow-up study also revealed that these children were more obese and insulin resistant as young adults. Furthermore, excessive gestational weight gain was linked to a higher BMI and skinfold measures in children at 3 years old, with a four-fold increased risk of having an overweight child. These findings highlight the significant long-term impact of maternal obesity and weight gain on child health [33].

These effects are often compounded by inflammation in adipose tissue, where exposure to a high-fat diet can induce the activation of inflammasomes and promote pro-inflammatory cytokine production, such as interleukin-1β (IL-1β). The offspring of HFD-fed dams exhibit a higher concentration of IL-1β in adipose tissue, as well as structural changes like crown-like structures, indicating inflammation [34,35]. This inflammation contributes to the development of insulin resistance, a precursor to type 2 diabetes, and can also lead to liver steatosis, as observed in both rodent models and human studies.

### Sex-Specific Effects of Maternal Diet on Offspring

Emerging research indicates that maternal diet quality can have sex-specific effects on offspring development, particularly through its influence on placental signaling. A study examined 108 pregnant women and assessed their dietary habits using the Healthy Eating Index (HEI), finding that maternal diet quality was associated with different patterns of placental signaling in male versus female offspring [36]. For female offspring, a higher maternal HEI score was linked to greater abundance of insulin and insulin-like growth factor 1 (IGF-1) receptors in the placenta. These receptors are crucial for regulating nutrient transport and promoting fetal growth, suggesting that a better-quality maternal diet may enhance signaling pathways that facilitate proper growth and metabolic function during pregnancy in female offspring.

In contrast, for male offspring, a higher maternal HEI was associated with increased activation of signaling pathways related to environmental stress, inflammation, growth factors, and intracellular kinases, including proteins like p38MAPK and JNK. Additionally, in males, a higher-quality maternal diet was linked to increased activation of mTORC1, a crucial cellular regulator of cellular homeostasis that controls cell growth, metabolism, autophagy, and protein synthesis. These findings suggest that maternal diet may influence growth-related processes through different molecular mechanisms in male versus female offspring [1,36].

## Metabolic Consequences of Preterm Birth in Offspring

### Preterm Birth and Development of Metabolic Disorders

Preterm birth, particularly in very low birth weight (VLBW) infants, is associated with an increased risk of metabolic disorders later in life. Studies have demonstrated that preterm infants have a higher risk of developing obesity, type 2 diabetes, and metabolic syndrome during childhood and into adulthood [37,38]. This increased susceptibility to metabolic dysfunction is thought to result from alterations in metabolic programming that occur due to the interruption of normal fetal development.

A study aimed to assess body composition changes in neonates in the neonatal intensive care unit (NICU) using air displacement plethysmography where the researchers measured body composition at two time points: 37.5 weeks and 41.0 weeks postmenstrual age. The results showed significant increases in weight and percentage body fat, with preterm infants having a higher mean body fat percentage than term infants. Abdominal girth increased, while mid-arm circumference decreased [39,40].

Birth disrupts the maternal–placental unit, causing endocrine and nutritional imbalances in preterm infants that are not seen in term-born infants. This withdrawal of maternal and placental hormones, such as IGF-1, leptin, thyroid hormones, and others, can lead to growth impairments and unfavorable neonatal outcomes, resembling a condition like “panhypopituitarism.” While proper, gestational age-adapted nutrition is crucial for optimal growth, it may not be sufficient alone. Research on the metabolic and endocrine disruptions in preterm infants, particularly involving hormones like IGF-1 and thyroid hormones, is needed to develop therapeutic approaches. Supplementing growth-promoting hormones, such as IGF-1 and thyroid hormones, may help prevent growth-related disorders and support development. However, the short- and long-term effects of these therapies need further exploration in clinical trials and animal models [41]. Very preterm infants (<32 weeks’ gestation) also exhibit poor postnatal growth relative to intrauterine growth, accumulating fat rapidly but having a significant deficit in fat-free mass. At term-equivalent age, these infants show increased body fat percentage compared to term-born infants, suggesting that rapid fat accretion post-birth may mask underlying deficiencies in fat-free mass [42].

Research has shown that preterm infants exhibit disturbances in glucose metabolism, with increased risk of insulin resistance and altered glucose handling. These metabolic alterations may persist into adulthood, contributing to the long-term health risks associated with preterm birth. Additionally, hyperglycemia, commonly observed in preterm infants, has been linked to adverse outcomes such as increased mortality and impaired neurodevelopment [43].

### Hormonal Profiles and Insulin Regulation in Preterm Infants

The hormonal profiles of preterm infants show significant disturbances that may contribute to their long-term metabolic risks. A study examined the hormonal concentrations and metabolic markers in very low birth weight infants at two time points: day of life 7 and postmenstrual age 36 weeks. The results revealed substantial hormonal changes over time, reflecting early disruptions in metabolic regulation.

At day of life 7, the infants exhibited elevated proinsulin and insulin concentrations compared to 36 weeks, with proinsulin levels decreasing from 42.6 pmol/L to 13.4 pmol/L, and insulin levels dropping from 183.8 pmol/L to 80.3 pmol/L [44]. These hormonal shifts indicate an initial insulin resistance and impaired insulin response in preterm infants, which may predispose them to metabolic dysfunction as they grow.

Furthermore, leptin, a hormone critical for regulating energy balance, showed a notable increase over time, from 0.11 μg/L at day of life 7 to 0.55 μg/L at postmenstrual age 36 weeks. Additionally, insulin sensitivity, measured using the HOMA2 index, was significantly lower at day of life 7 (HOMA index of 3.6) compared to 36 weeks (HOMA index of 1.6), reinforcing the notion that early metabolic stress in preterm infants may lead to lasting insulin resistance [44].

### Long-term Cardiovascular and Metabolic Effects

Preterm birth and neonatal hyperglycemia have been linked to various long-term health complications, including effects on cardiovascular development. Research suggests that these early-life factors may contribute to structural heart changes and other metabolic abnormalities later in life [45]. Preterm infants, especially those born before 35 weeks, exhibit early signs of endothelial dysfunction, a key marker of cardiovascular disease. This dysfunction, along with altered body composition (lower lean mass and increased body fat), predisposes them to conditions like type 2 diabetes and cardiovascular disease in adulthood [46].

A study found that longer periods of hyperglycemia during the neonatal period were associated with increased cardiac wall thickness, including the interventricular septum and left ventricle posterior wall, as well as changes in left atrial morphology in children born extremely preterm. Additionally, neonatal lipid and carbohydrate intakes were linked to specific alterations in cardiovascular structure, such as increased aortic diameter and smaller aortic annulus [39].

Moreover, the potential intergenerational effects of preterm birth on metabolic function have been investigated. A study explored insulin sensitivity in adults who were born preterm, as well as in their children. The study found that adults born preterm had similar fasting plasma glucose concentrations, but significantly higher insulin levels compared to those born at term. Insulin sensitivity was 47% lower in adults born preterm, and this reduction remained significant even after adjusting for factors like age, sex, BMI, and antenatal steroid exposure [40].

**Figure 1:**
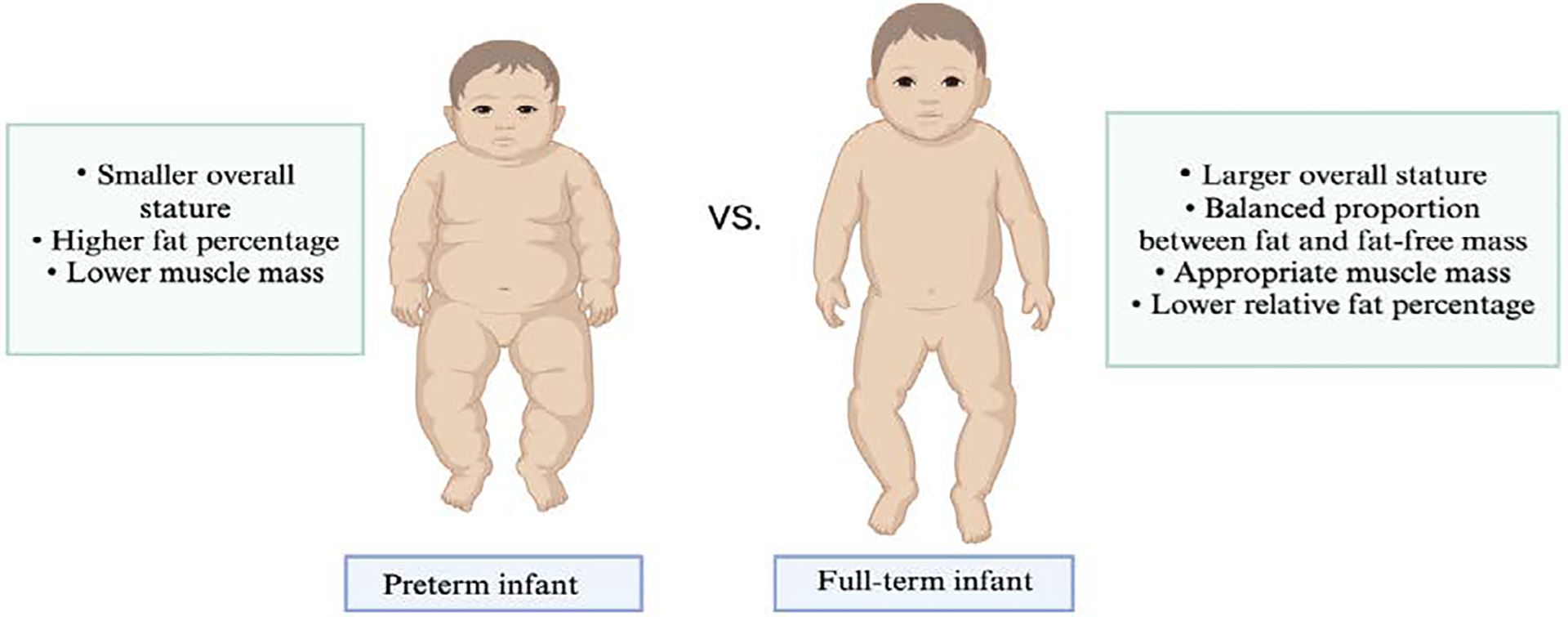
Comparative Body Composition Visualization of Preterm and Full-Term Infants. This side-by-side anatomical representation illustrates the paradoxical body composition phenotype observed in preterm infants compared to their full-term counterparts. Despite their smaller overall size, preterm infants (left) demonstrate proportionally higher adiposity with a greater percentage of total body weight as fat, particularly in subcutaneous deposits. Conversely, preterm infants exhibit significantly reduced fat-free mass, reflected in diminished muscle volume and bone density. Full-term infants (right) display more balanced body composition with appropriate fat-to-lean mass ratios consistent with optimal metabolic development.

Adults born preterm also showed a compensatory increase in both first- and second-phase insulin secretion, though there was no evidence of a defect in β-cell function. Interestingly, while children of parents born preterm exhibited similar glucose metabolism parameters to those whose parents were born at term, insulin sensitivity in these children was positively correlated with the insulin sensitivity of the preterm-born parent (Figure 1). This suggests potential intergenerational effects on metabolic function, with possible genetic or environmental factors influencing metabolic outcomes in offspring [40].

Although majority of adults born pre-term survive without significant co-morbidities, the numbers are lower than those born full-term. These findings are consistent for men and women. Sibling analyses suggested that genetic and environment factors had little effect on this discrepancy [47]. A study examining pulmonary vascular function in young adults born preterm found that, despite having no history of adult cardiopulmonary disease, these individuals exhibited elevated resting pulmonary arterial pressures (PAP) and increased pulmonary vascular resistance. Notably, 45% of the preterm participants had PAP levels considered abnormal, with some showing evidence of early right ventricular dysfunction during exercise. Although the hypoxic vasoconstrictor response was intact, these individuals demonstrated a stiffer, less-recruitable pulmonary vascular bed and an impaired ability to increase cardiac index or right ventricular stroke work during physical stress. These findings suggest that even in the absence of overt cardiopulmonary disease, preterm birth is associated with early pulmonary vascular changes and right ventricular dysfunction, highlighting the need for further studies on early intervention to prevent long-term complications in this high-risk group [48,49].

## Combined Effects of Maternal UPF Consumption and Preterm Birth on Offspring Metabolism

Maternal dietary patterns, especially the consumption of ultra-processed foods (UPFs), are known to influence both maternal and fetal health, contributing to adverse birth outcomes, including preterm birth, gestational weight gain, and diabetes. These outcomes can have a lasting impact on offspring metabolism and development. A study on beverage intake during pregnancy found a significant relationship between higher intake of cultured-milk drinks and an increased risk of developing gestational diabetes mellitus (GDM), which is linked to adverse birth outcomes such as preterm birth. Women with higher intake of sugary beverages and unhealthy foods during the first trimester were more likely to develop GDM and have subsequent complications [50–52].

Infections and inflammation play a central role in preterm birth, as pathogens can trigger inflammatory responses leading to uterine contractions and preterm labor. This response involves the release of mediators like prostaglandins and nitric oxide, which are key in labor initiation [53–56]. Inflammation is also a critical factor in conditions such as atopic dermatitis. A prospective cohort study found that higher maternal UPF intake during pregnancy was strongly associated with an increased risk of infantile atopic dermatitis, potentially due to the pro-inflammatory effects of UPFs [53]. The link between UPF consumption and inflammation could contribute to both preterm birth and immune dysfunction in the offspring, as both are rooted in immune system overactivation [57].

Greater UPF intake during pregnancy has been associated with increased gestational weight gain, postpartum weight retention, and elevated C-reactive protein (CRP) levels, a marker of inflammation. This study revealed that more than half of pregnant women’s daily energy intake came from UPFs, aligning with trends seen in non-pregnant populations in the United States. However, UPF consumption was not significantly linked to maternal cardiometabolic markers or infant weight-for-length. The study emphasized the need for further research into the mechanisms of UPF impact, such as increased total energy, added sugars, nutrient displacement, and changes to the gut microbiome [58,59].

Research also highlights the relationship between UPF consumption and poor glycemic control during pregnancy. A study on pregnant women with preexisting diabetes showed that regular consumption of UPFs was associated with increased maternal blood glucose levels and gestational weight gain. The trend of shifting from traditional diets to UPF-based diets, exacerbated by socio-economic factors, increases the risk of adverse pregnancy outcomes such as hypertensive disorders, inadequate glycemic control, and macrosomia [60].

Excessive gestational weight gain, particularly when linked to UPF consumption, increases the risk of hypertensive pregnancy syndromes, such as preeclampsia. Hypertensive disorders complicate 8–10% of pregnancies and are responsible for 8–10% of preterm births, with obesity being a major risk factor. Preeclampsia is linked to an increased risk of preterm births, especially in women with diabetes. The condition is characterized by hypertension, proteinuria, and organ dysfunction, typically occurring after 20 weeks’ gestation. Clinical risk factors for preeclampsia include a history of the condition, chronic hypertension, diabetes, obesity, and advanced maternal age [61]. However, predicting preeclampsia remains challenging due to the lack of specific biomarkers [62–65].

Increased consumption of UPFs not only increases the risk of preterm birth but also has significant implications for fetal metabolism. The influence of maternal diet on fetal metabolic programming is gaining attention, as it can shape the metabolic health and development of the fetus, potentially leading to long-term consequences for offspring health [66,67]. A recent study provided insights into fetal tissue metabolism during mid-to-late gestation, comparing the effects of maternal euglycemia and hyperglycemia. The study found that fetuses from hyperglycemic dams exhibited elevated levels of sorbitol, a glucose-derived toxic metabolite, suggesting that fetal tissues are not shielded from sorbitol accumulation, which can cause tissue damage in diabetic conditions [68–70]. Additionally, altered amino acid levels, including reduced GABA, were observed in the fetal brain, potentially contributing to congenital brain defects. The study also highlighted changes in fetal nutrient sourcing, revealing that fetal liver and brain metabolism showed increased glucose dependence in the context of maternal hyperglycemia. While euglycemic fetuses derive carbon backbones flexibly from glycogen breakdown or lactate via gluconeogenesis, hyperglycemic fetuses displayed increased reliance on glucose. This shift underscores the metabolic plasticity of fetal tissues when exposed to altered nutrient environments [70].

Another interesting finding from the study was the significant accumulation of histamine and histidine-derived metabolites in fetal tissues and maternal plasma during late gestation. Elevated histamine levels have been associated with preterm labor, further suggesting that maternal UPF consumption could contribute to preterm delivery via metabolic disruptions [70].

**Figure 2:**
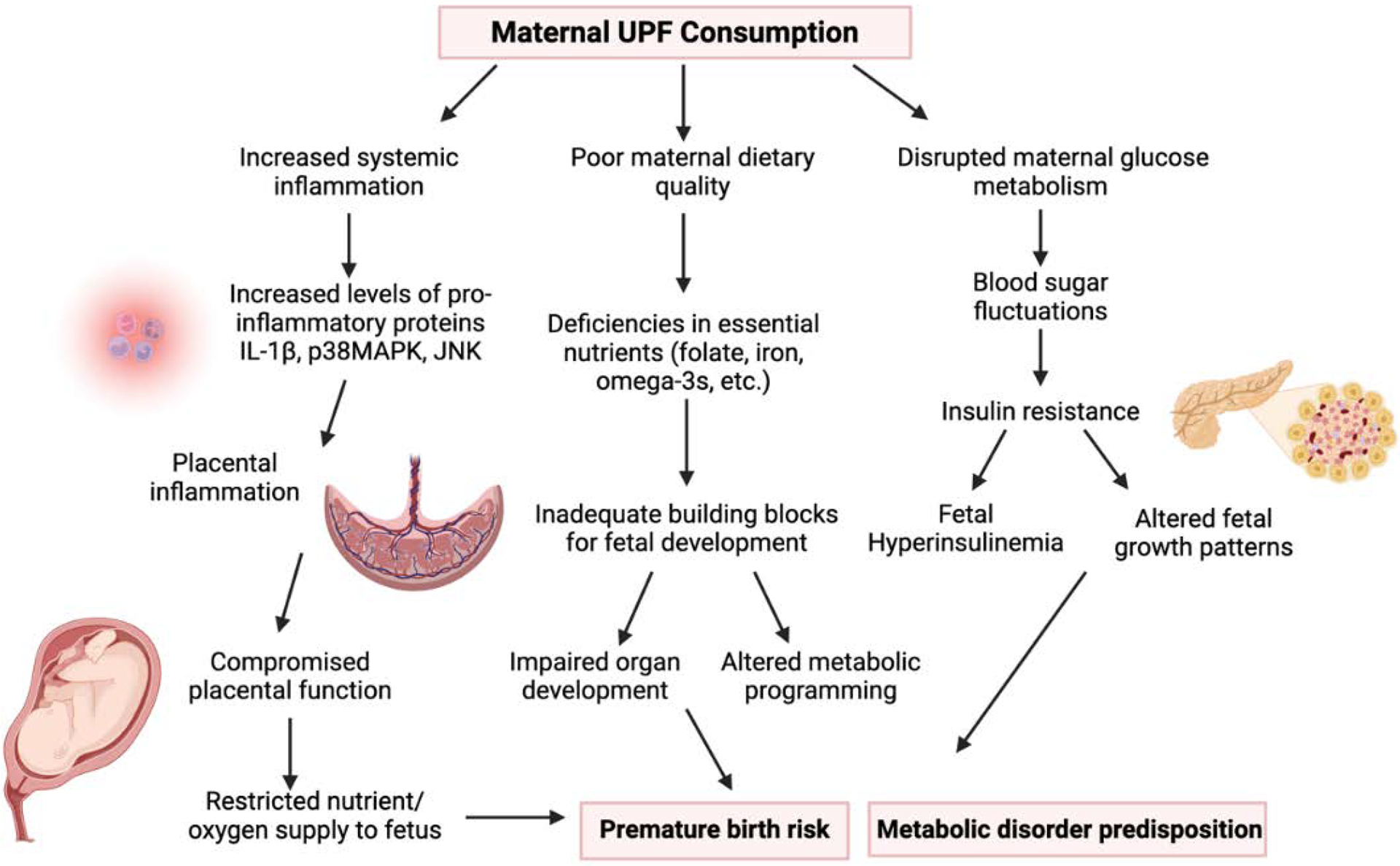
Maternal Ultra-Processed Food (UPF) Consumption Pathways and Fetal Development Outcomes. The three primary mechanistic pathways (inflammation, nutrient imbalance, and hormonal disruption) through which maternal consumption of ultra-processed foods may adversely affect fetal development. This multi-mechanism model provides a framework for understanding the transgenerational impacts of modern dietary patterns on developmental outcomes.

The combined effects of maternal UPF consumption and preterm birth represent a critical area of research, particularly in understanding how maternal diet impacts fetal metabolism and long-term offspring health. Evidence indicates that not only does UPF consumption increase the risk of preterm birth, but it also disrupts fetal metabolic pathways, leading to the accumulation of harmful metabolites and altering nutrient sourcing (Figure 2). These findings emphasize the need for continued investigation into the mechanistic links between maternal diet, fetal metabolism, and pregnancy outcomes.

## Public Health Implications

### Importance of Maternal Nutrition During Pregnancy

The evidence presented in this review underscores the critical importance of maternal nutrition during pregnancy for both preventing preterm birth and reducing the risk of metabolic disorders in offspring. Promoting healthy, nutrient-dense diets for pregnant women is essential for optimizing fetal development and reducing the risk of adverse birth outcomes.

A study examining the relationship between maternal socioeconomic status and nutrition, focusing on underweight and overweight/obesity prevalence in women across 49 countries confirmed that higher national income levels are linked to lower rates of underweight and higher rates of overweight/obesity, with a non-linear association, showing steeper increases in obesity at higher levels. The study reveals that the nutrition transition affects women differently across socioeconomic groups, with poorer women in low-income countries more likely to be underweight, while wealthier women in middle-income countries are driving obesity rates. These findings suggest the need for targeted interventions for underweight women and preventive strategies for wealthier women. The study underscores the importance of disaggregating data by socioeconomic status and regularly monitoring nutrition trends to inform equitable public health strategies [71].

Malnutrition, including undernutrition, overweight and obesity, and micronutrient deficiencies, continues to affect millions of women and children, particularly in low- and middle-income countries (LMICs). Supplementary food provision in food-insecure settings and community-based approaches to manage acute malnutrition have also shown positive results. Emerging interventions, such as small-quantity lipid-based nutrient supplements for children aged 6–23 months, have had positive effects on growth. Integrated interventions, including diet, exercise, and behavioral therapy, are effective for preventing and managing childhood obesity, though evidence from LMICs is limited. Additionally, indirect nutrition strategies, such as malaria prevention and improved water, sanitation, and hygiene, contribute to nutritional benefits [72,73]. However, recent global challenges, including the COVID-19 pandemic, conflicts, climate change, economic downturns, and persistent inequality, have exacerbated food insecurity and malnutrition, particularly in LMICs. These factors, along with the rising cost of healthy diets, are key barriers to achieving food security. Despite setbacks, the pandemic has revealed vulnerabilities that, if addressed, could lead to transformative food system solutions [74,75].

Between 2008 and 2018, approximately 18.6% of pregnant women enrolled in Special supplemental nutrition program for women, infants and children (WIC) had anemia, with variations by state, race, and trimester. Anemia prevalence among African American pregnant women and those assessed in the third trimester was categorized as a moderate public health problem. WIC data allows for monitoring anemia trends and targeting interventions for low-income pregnant women at higher risk. Despite some limitations, WIC’s anemia surveillance helps identify women needing additional nutritional support [76–79].

Maternal undernutrition remains a significant public health issue, with disparities in underweight, anemia, and micronutrient deficiencies within the United States. These disparities are influenced by factors such as access to health services, women’s status, food insecurity, and social, economic, and political contexts. Nutrition interventions before and during pregnancy, such as balanced-energy protein supplements and multiple micronutrient supplements, have shown positive effects on birth outcomes. However, further research is needed to address preconception nutrition, long-term effects on offspring, and to focus on implementation science and equity to reduce maternal undernutrition disparities [80]. Obesity (BMI of 30.0 and over) has increased in the United States in recent decades, with variations based on age, race, Hispanic origin, and socioeconomic status. Maternal obesity is associated with adverse health outcomes for both mothers and infants, including gestational diabetes, hypertension, preeclampsia, and preterm delivery [81].

### Interventions for High-Risk Populations

Maternal nutrition interventions are crucial for improving birth outcomes, particularly for high-risk populations, including women with inadequate nutrition, low socioeconomic status, or limited access to healthcare. These interventions are designed to address nutritional deficiencies and promote healthy maternal behaviors before and during pregnancy, which are key determinants of fetal development and birth weight [82]. High-risk populations, such as those in low-income settings or with limited access to nutritious food, are more vulnerable to complications like low birth weight (LBW), preterm birth, and other adverse outcomes. Integrated maternal nutrition intervention packages, which combine education, dietary supplementation, and socioeconomic support, have shown promise in improving maternal nutritional status and reducing LBW. These multi-sectoral interventions can help address underlying causes like poor diet, inadequate weight gain, and poor maternal health, ultimately leading to better birth outcomes [83,84].

Evidence from randomized controlled trials (RCTs) indicates that interventions to improve maternal nutrition, such as blanket supplementation with balanced protein-energy (BPE), multiple micronutrients (MMN), lipid-based nutrient supplements (LNS), or omega-3 fatty acids (Ω3FA), can reduce the prevalence of low birth weight (LBW) and related adverse birth outcomes, such as preterm birth (PTB), small for gestational age (SGA), and stillbirth [84]. Balanced protein-energy supplementation specifically decreases the risk of stillbirth, while high-dose calcium supplementation and dietary education may reduce LBW prevalence, although the effects of low-dose calcium remain inconclusive. The efficacy of weight gain-promoting interventions has yet to be fully established, particularly in targeting women with inadequate weight gain. While universal nutrient supplementation is effective, more research is needed to identify the most cost-effective approaches for both universal and targeted interventions. A holistic approach integrating nutrition with social, environmental, and medical support is essential for improving maternal and birth outcomes [85].

The consumption of ultra-processed foods (UPFs) has been steadily increasing, with recent studies highlighting concerning trends in both developed and developing countries [86–88]. In many places, UPFs are not only becoming more prevalent but also more affordable and convenient, making them the go-to option for many families, especially those with limited time or financial resources [89,90]. This rise in UPF consumption is particularly alarming among pregnant women, as these foods are linked to adverse health outcomes for both mothers and their fetuses. For expectant mothers, the nutritional quality of their diet directly impacts fetal development, making it crucial to reduce UPF consumption during pregnancy [91]. Given that UPFs are often cheaper and more accessible than fresh, whole foods, addressing this issue will require comprehensive strategies, including behavioral interventions, educational programs, and policy changes that make healthier options more affordable and appealing. Reducing UPF consumption, especially among mothers, is essential for improving maternal and fetal health outcomes and can contribute to healthier future generations [92,93].

A study tested a behavioral intervention to reduce ultra-processed food (UPF) intake in the United States, showing promising results in both feasibility and effectiveness. Participants reduced their daily UPF consumption by approximately 24%, with a marked decrease in overall calorie intake, leading to an average weight loss of 3.5 kg over the 8-week program. The intervention also resulted in significant improvements in dietary quality, such as reduced intake of saturated fat, added sugars, and sodium, without compromising fruit and vegetable consumption. Participants reported physical benefits like better skin, reduced swelling, and improved mood, further emphasizing the positive impact of reducing UPF intake [94]. While the intervention was well-received, challenges such as unsupportive household members and the difficulty in scaling financial support were noted. Despite these limitations, the results suggest that reducing UPF intake is an effective and acceptable approach for improving physical and mental health [95]. However, further research with larger, more diverse populations and longer follow-ups is necessary, along with policy changes to support broader reductions in UPF consumption.

A study conducted in Brazil, investigated the impact of an educational intervention on reducing ultra-processed food (UPF) consumption during pregnancy. Pregnant women with low obstetric risk were assigned to either an intervention group (n=181) or a control group (n=172). Health professionals in the intervention group were trained to encourage healthy eating practices, including consuming fruits, vegetables, beans, and limiting soft drinks and industrial cookies during prenatal care. Dietary recalls were taken at two trimesters to assess food consumption, with foods classified according to the Nova classification, which is a framework for grouping edible substances based on the extent and purpose of the applied food processing. Results showed that a quarter of the energy consumed by the participants came from ultra-processed foods, and the intervention reduced the percentage of energy from UPFs by 4.6 points between the first and second trimesters (p=0.015), though no effect was observed in the third trimester. The study concluded that training healthcare professionals to promote healthy eating practices is an effective and sustainable approach to reducing UPF consumption during pregnancy [96,97].

A community-based initiative known as the UnProcessed Pantry Project demonstrated that the emergency food system, particularly food pantries, can play a crucial role in improving the dietary quality and health of food-insecure populations. By shifting the focus from ultra-processed foods (UPFs) to unprocessed, nutrient-dense foods, the unprocessed pantry project, UP3, significantly improved participants’ dietary quality, as measured by the Healthy Eating Index (HEI), and led to improvements in biomarkers such as BMI, cholesterol, and waist circumference. The 16-week intervention, which included both access to healthier foods and educational components, showed that small dietary changes can have meaningful health benefits, even in the short term. Participants reported increased engagement with healthy eating practices and a better understanding of the impact of UPF on their health. However, some participants noted the challenge of the low cost of UPFs, highlighting the need for the emergency food system to provide more unprocessed food options. The success of the intervention was largely due to strong partnerships and the involvement of stakeholders, ensuring a tailored, participatory approach. This model shows that food pantries can be an effective platform for addressing both food insecurity and the risk of non-communicable diseases [98–100].

### Policy Recommendations

The United States policy efforts to address ultra-processed foods (UPFs) are relatively recent and limited. While many policies mention UPFs in relation to promoting healthy diets, few directly target them. Common policy efforts focus on nutrition education for children and improving the food environment by incentivizing small retailers in low-resource areas to stock healthier foods. The issue of food pricing is also a significant concern, as UPFs tend to be much cheaper per calorie compared to unprocessed foods, making them more accessible but potentially contributing to nutrition insecurity in low-income households [101].

The United States government has taken some steps, such as the 2015–2020 Dietary Guidelines for Americans (DGAs), which included definitions for processed meats, and New York City’s successful removal of processed meats from public schools. However, the 2025–2030 DGA Advisory Committee is only examining UPFs in relation to body weight, with no clear indication of whether they will address broader issues like diet quality. One state-level initiative, Massachusetts’ school food bill, has taken a more direct approach by combining food categories and processing levels to identify UPFs. Similarly, Brazil has implemented policies that prohibit UPFs in schools, which could serve as a model for United States policy.

These developments highlight the challenges of defining and regulating UPFs. The experience of the U.S. National School Lunch Program shows that nutrient-based guidelines can lead to the reformulation of UPFs without necessarily improving their nutritional quality. To address UPFs effectively, policies should combine both food categories and processing levels, with a focus on non-staple foods, which may face less opposition from the food industry. Such an approach could help reduce the negative impact of UPFs on public health, particularly for vulnerable populations [101–104].

A study highlighted significant gaps in national and global policies regarding maternal nutrition interventions (MNIs) within antenatal care (ANC) services, including the lack of detailed protocols for micronutrient supplementation, weight gain counseling, dietary advice, and breastfeeding support. These gaps stemmed from insufficient specificity in national ANC guidelines, inadequate alignment with global recommendations, and a lack of accountability for the quality and coverage of nutrition interventions [106,107]. The study suggests improving coordination between nutrition and maternal health units, developing clear operational guidelines for ANC staff, and consolidating nutrition protocols in accessible policy documents. It also called for better clarity in global guidelines to support country-specific adaptations, addressing barriers such as micronutrient stock-outs, knowledge gaps, and insufficient care-seeking. Social factors, including family support and community engagement, were identified as crucial to improving adherence to nutrition practices, and the study emphasized the need for sustained individual and community-level interventions, including repeated counseling throughout pregnancy. Ultimately, it provided critical evidence and recommendations for filling gaps in ANC services to enhance the effectiveness of nutrition interventions during pregnancy [105,107,108].

In conclusion, both the United States policy efforts to address ultra-processed foods (UPFs) and the global and national policies surrounding maternal nutrition interventions (MNIs) reveal significant challenges and gaps in effectively promoting public health. While the United States has made some strides in addressing UPFs, particularly through initiatives like the Dietary Guidelines for Americans [109] and state-level bills, there is still a lack of comprehensive regulation and focus on the broader issues of diet quality. Similarly, the study on maternal nutrition highlights the insufficient specificity and coordination of ANC services, emphasizing the need for clearer guidelines and better integration of nutrition and maternal health strategies. Both areas demonstrate the importance of clear, targeted policy frameworks that not only address the direct issues at hand but also consider social, economic, and systemic barriers to success. Effective policy interventions must prioritize accessible, evidence-based strategies, combine regulatory approaches with community engagement, and continuously adapt to emerging challenges to improve health outcomes for vulnerable populations [110,111].

## Limitations and Future Research Directions

The current body of research on maternal ultra-processed food (UPF) consumption, preterm birth, and offspring metabolic health has several key limitations. These include the lack of studies that examine the combined effects of maternal UPF intake and preterm birth on offspring outcomes. Many studies fail to account for confounding factors like socioeconomic status, genetics, and environmental exposures, which can influence both maternal diet and offspring health. In addition, there is inconsistency in how UPFs are defined and categorized, making it difficult to compare results across studies. Most research also suffers from short follow-up periods, relying on self-reported dietary data, which is prone to recall bias and underreporting, especially for socially undesirable foods like UPFs.

To address these limitations, future research should focus on several key areas. Longitudinal studies with extended follow-up periods are necessary to assess the long-term metabolic consequences of maternal UPF consumption and preterm birth on offspring. Further investigation into the biological mechanisms, including epigenetic modifications, inflammation, and hormonal regulation, is needed to better understand the links between these factors. Intervention studies should explore how dietary changes during pregnancy can reduce UPF consumption and improve metabolic health outcomes for offspring. There is also a need for sex-specific studies, as evidence suggests that males and females may be differentially affected by maternal diet.

Additionally, research should explore the transgenerational effects of maternal UPF consumption and preterm birth on metabolic health in subsequent generations. Personalized approaches to nutrition, considering individual risk factors such as pre-existing metabolic conditions, genetic predispositions, and environmental exposures, should be developed to better guide dietary recommendations for pregnant women. This approach would help to create more effective, targeted strategies to mitigate the potential negative impacts of UPFs on maternal and offspring health.

## Conclusion

This review highlights the significant impact of maternal ultra-processed food consumption and preterm birth on the development of metabolic disorders in offspring. The evidence suggests that both factors independently contribute to altered metabolic programming in children, with potential synergistic effects when they co-occur [111].

Maternal UPF consumption during pregnancy can disrupt normal fetal development through various mechanisms, including nutrient imbalances, inflammation, and hormonal dysregulation. These disruptions may lead to preterm birth and subsequent metabolic disorders in offspring. Similarly, preterm birth itself is associated with alterations in metabolic regulation, potentially predisposing children to obesity, insulin resistance, and cardiovascular disease later in life.

The public health implications of these findings are substantial, highlighting the need for targeted interventions to reduce UPF consumption among pregnant women and improve prenatal care to reduce preterm birth rates. Future research should focus on elucidating the mechanisms underlying the combined effects of maternal UPF consumption and preterm birth on offspring metabolic health, as well as developing effective interventions to mitigate these risks [112–115].

To address the challenges associated with maternal ultra-processed food (UPF) consumption and its effects on preterm birth and offspring metabolic health, public health interventions must take a multi-faceted approach. Public health strategies need to be comprehensive, incorporating both prevention and intervention measures at various levels, from individual behavior change to broader policy reforms. The first step in this approach is to raise awareness among the public, especially pregnant women and their families, about the risks associated with UPF consumption during pregnancy. Educational campaigns could focus on the importance of a balanced diet and the potential long-term consequences of poor maternal nutrition on both the mother and her offspring. This can be achieved through digital media, public service announcements, healthcare settings, and community outreach programs [113–115].

Moreover, policies need to be developed and expanded to regulate the marketing, availability, and affordability of UPFs, especially in low-income communities. Public policies could focus on improving food environments by incentivizing retailers to stock healthier, minimally processed foods, particularly in areas where access to fresh produce is limited. Additionally, governments should consider implementing food labeling laws that make it easier for consumers to identify and avoid UPFs. Economic policies that make unprocessed or minimally processed foods more affordable than UPFs can also help reduce consumption, particularly for low-income families who may rely on cheaper, highly processed foods. Policies that restrict the marketing of unhealthy, ultra-processed foods to pregnant women and children, especially in schools or healthcare settings, can also be effective in reducing exposure to harmful dietary patterns.

In parallel, healthcare providers and public health systems must prioritize education on maternal nutrition, ensuring that prenatal care programs not only screen for UPF consumption but also provide counseling on healthier dietary choices. Healthcare professionals, including obstetricians, dietitians, and community health workers, must be trained to recognize the signs of poor maternal diet and be equipped with the knowledge to deliver personalized nutrition advice tailored to the needs of individual women. Maternal education programs should emphasize the benefits of a nutrient-dense diet and explore strategies to reduce reliance on UPFs during pregnancy. This could include training on cooking skills, meal planning, and understanding food labels, as well as addressing cultural beliefs and social norms around food that may influence dietary habits. Engaging family members, especially partners, in these programs can help ensure that pregnant women receive the necessary support from their households to make healthier choices, and community-based initiatives could foster collective behavior change.

By addressing these challenges through a combination of individual-level interventions, community-based programs, and policy-level changes, we can work towards improving maternal nutrition during pregnancy, reducing the incidence of preterm birth, and ultimately enhancing the metabolic health of future generations.

## Key Points, Challenges and Outstanding questions

- Ultra-processed food (UPF) consumption by pregnant women significantly impacts fetal development and metabolic programming, potentially predisposing offspring to obesity, insulin resistance, and other metabolic disorders later in life.
- Preterm birth disrupts normal metabolic programming, with preterm infants showing disturbances in glucose metabolism, hormonal profiles, and body composition (higher body fat percentage but lower lean mass) that persist into adulthood.
- The quality of maternal diet during pregnancy has a greater impact on fetal growth and development than maternal pre-pregnancy body mass index or weight gain, highlighting the importance of nutrient-dense foods over caloric intake alone.
- While both maternal UPF consumption and preterm birth independently increased risk for metabolic disorders in offspring, their combined effects may be synergistic, compounding long-term health risks.
- Most studies have short follow-up periods, rely on self-reported dietary data prone to recall bias, and fail to account for confounding factors like socioeconomic status and environmental exposures.
- There are significant gaps in both national and global policies regarding maternal nutrition interventions, including insufficient specificity in guidelines and lack of accountability for quality and coverage of nutrition interventions.
- UPFs are often cheaper and more accessible than fresh foods, creating economic barriers to improving maternal nutrition, especially in low-income populations.
- What are the precise biological mechanisms through which maternal UPF consumption and preterm birth together influence metabolic programming in offspring?
- To what extent do the metabolic effects of maternal UPF consumption and preterm birth extend to subsequent generations, and what epigenetic mechanisms might be involved?
- What specific intervention strategies are most effective in reducing UPF consumption during pregnancy across different socioeconomic and cultural contexts?
